# An assessment of the quality indicators of operative and non-operative times in a public university hospital

**DOI:** 10.1590/S1679-45082015GS3289

**Published:** 2015

**Authors:** Altair da Silva Costa, Luiz Eduardo Villaça Leão, Maykon Anderson Pires de Novais, Paola Zucchi

**Affiliations:** 1 Hospital Israelita Albert Einstein, São Paulo, SP, Brazil.; 2 Universidade Federal de São Paulo, São Paulo, SP, Brazil.

**Keywords:** Quality indicators, health care, Operating rooms, Operative time

## Abstract

**Objective:**

To assess the operative time indicators in a public university hospital.

**Methods:**

A descriptive cross-sectional study was conducted using data from operating room database. The sample was obtained from January 2011 to January 2012. The operations performed in sequence in the same operating room, between 7:00 am and 5:00 pm, elective or emergency, were included. The procedures with incomplete data in the system were excluded, as well as the operations performed after 5:00 pm or on weekends or holidays.

**Results:**

We measured the operative and non-operative time of 8,420 operations. The operative time (mean and standard deviation) of anesthesias and operations were 177.6±110 and 129.8±97.1 minutes, respectively. The total time of the patient in operative room (mean and standard deviation) was 196.8±113.2. The non-operative time,* e.g.*, between the arrival of the patient and the onset of anesthesia was 14.3±17.3 minutes. The time to set the next patient in operating room was 119.8±79.6 minutes. Our total non-operative time was 155 minutes.

**Conclusion:**

Delays frequently occurred in our operating room and had a major effect on patient flow and resource utilization. The non-operative time was longer than the operative time. It is possible to increase the operating room capacity by management and training of the professionals involved. The indicators provided a tool to improve operating room efficiency.

## INTRODUCTION

In the United States, 15 million surgical procedures have been done (495 procedures/10 thousand inhabitants), with hospital costs of approximately US$ 171 billion. Surgical patients generate greater costs than do clinical patients, US$ 2,900.00 and US$ 1,400.00, respectively, per day of hospitalization. The operating room (OR) should be efficient and optimized, as well as the available time and resources.^(1-3)^

The OR is one of the most complex structures of the hospital system: it functions 24 hours a day, seven days a week, and there is a difference in complexity in terms of equipment and procedures. It also needs several classes of professionals – administrator, physicians, nurses, information technology, pharmacists, cleaning employees, among others. Logistics of the material should be perfect, since its absence has implications in the quality of the procedure and its results. Additionally, there is the presence of biological material and the patient’s risk of life, which are always present.^(4,5)^

The OR is also the intersection of various sectors of the hospital, such as intensive care, inpatients units, emergency room, laboratory, and imaging diagnostics. The adequate operation also depends on the physical structure, new technologies, appropriate materials and pieces of equipment managed by skilled personnel who are trained and competent.^(6,7)^ It is a highly distinguished environment, and for this reason waste of time and material should be minimal. A poorly efficient OR generates more risks and waste to the hospital, patients, and employees. The OR should be the most efficient sector of the hospital, with processes under constant control and optimization.^(8)^

The processes represent tasks being executed, direct or indirectly related to the procedure to be carried out.^(9,10)^ In order to measure the processes, data are collected from forms, and thus, productivity indicators are created, such as cancelled surgeries, late professionals, number of surgeries per OR or per day, or lack of adequate material in the OR. Other time indicators are used in hospital services and in the OR, such as time of anesthesia and time to prepare the OR.^(11,12)^

Preparation of the OR, the turnover time, includes patient removal from the OR, cleaning, preparation of necessary instruments and materials, presence of professionals at the site (nurses, physicians, and technicians), and entrance of the patient into the OR.^(4,10,13-15)^ The ideal OR preparation time between one operation and another can be classified as of high performance if up to 25 minutes; of medium performance if between 25 and 40 minutes; not good enough if more than 40 minutes.^(4,10,13-15)^

The Surgery Management Improvement Group, from Michigan, United States, published the ideal time for each interval.^(14)^ A program was developed to optimize the time in the OR and measure the impact after implementation. The useful time was calculated for each surgical suite, its ideal performance, and thus, the potential for improvement was determined. The time measured between the entrance of the patient into the OR and beginning of the incision was 45.5 minutes. Ideal performance would be 19 minutes, and the potential for improvement was 26.5 minutes per operation. Another example was the preparation of the OR between one procedure and another; the time measured was 34.8 minutes, ideal performance was 23.2 minutes, and the potential for improvement was 11.6 minutes.^(14)^

Time management in the OR deserves to be emphasized. Its organization (or lack of), the cost of an idle OR, and the exhaustion of the professionals are examples of wasted time and resources that affect the patient, family members, the hospital, and the professionals.^(11,12)^

## OBJECTIVE

To evaluate the time indicators of the operating room of a university hospital, to improve its efficiency.

## METHODS

“Surgery” is defined as the branch of medicine dedicated to the treatment of diseases, lesions, or deformities by manual processes, called operations or surgical interventions. Surgery is a skill to be learned, studied, and taught. The treatment and the act in itself are called the “operation”. The surgeon performs an operation and studies or teaches surgery.

A cross-sectional descriptive study was conducted based on the database from the information technology system of the OR of the university hospital of the *Universidade Federal de São Paulo*. The sample was composed of operations carried out during the period from January 2011 to January 2012. The inclusions criterias were: surgical interventions performed in sequence, in the same OR, during the routine working hours, between 7:00 am and 5:00 pm, elective or urgent. The exclusions criterias were: procedures with incomplete data in the system, operations that began after 5:00 pm, or were performed over the weekends or on holidays, because on these periods the staff was reduced. The operating center of the university hospital was composed, at the time, of 19 actives ORs. The process that occurred in the OR was divided according to the following steps to collect the respective variables: 1) time of the patient in the OR; 2) time of duration of anesthesia; 3) time of duration of the operation; 4) time between the entrance of the patient into the OR and the beginning of anesthesia; 5) time between the beginning of anesthesia and the start of the surgery; 6) time between the end of the surgery and the end of the anesthesia; 7) time between the end of anesthesia and exit from the OR; 8) time until entrance of the next patient into the OR (turnover) – random sample; 9) time between 7:00 am and entrance of the first patient of the day – random sample. To analyze these last two indicators (the OR preparation time (turnover) and the entrance of the first patient of the day into the OR), the data was obtained by sampling and the individual calculation of each operation was performed. Six OR were randomly selected to achieve a representivity of at least 10% of the operations.

The first surgery of the day was not subject to the unpredictability of the others, and therefore, it was an important quality indicator of time and process. It reflected the organization of the sector.

The project was approved by the Research Ethics Committee of the organization, with official report number 165,292/2012, CAAE: 07233312.9.0000.5505, and authorized by the coordinator of the hospital’s operating center. The use of the Informed Consent Form was waived because of the research method applied.

## RESULTS

A total of 12,114 procedures were performed from January 2011 to January 2012. Of these, 8,420 (69.5%) operations were included in the study, and 3,694 (30.5%) procedures were excluded due to incomplete data. There were 4,600 (55.3%) elective operations and 3,760 (44.7%) urgent interventions.

It was analyzed 990 operations (11.7% of total) to calculate the turnover time and the time between 7:00 am and entrance of the first patient of the day, in six aleatory ORs.

The operating time ranged from zero minutes to 14.5 hours. The complexity was also very diverse. It varied from procedures such a catheter removal, nevus resections, up to organs transplants. Practically, all surgical specialties were included in the procedures analyzed: cardiac surgery, vascular surgery, neurosurgery, general surgery, gastric surgery, otolaryngology, head and neck, chest surgery, gynecology, ophthalmology, among others.

Table 1 shows the times related to permanence of the patient in the OR, anesthesia, and the surgery.

**Table 1:** Time of stay of the patient in the operating room, time of anesthesia, and operative time for 8,420 procedures

	Mean ± standard deviation	Median	Minimum-Maximum
Patient in operating room	196.8±113.2	177	12-900
Anesthesia	177.6±110	159	3-897
Surgery	129.8±97.1	111	0-843

Values expressed in minutes.

The time interval indicators showed what occurs between the procedures in the non-operative time, how much time was necessary between the end of one step and the start of the next (Table 2).

**Table 2:** Time between stages of the surgery in 8,420 procedures

	Mean ± standard deviation	Median	Minimum-Maximum
Entry into the operating room – start of anesthesia	12.3±17.3	5	0-93
Start of anesthesia – start of surgery	36±21.5	39	0-180
End of surgery – end of anesthesia	24.3±23	15	0-276
End of anesthesia – exit the operating room	14.7±22.5	5	0-258

Values expressed in minutes.

Of the 990 operations analyzed, there was a mean of approximately two hours for preparation of the OR (turnover) and entrance of the next patient, with a maximum time of almost eight hours. The mean with standard deviation of the time interval between the exit of the patient from the OR and entrance of the next patient into the OR was 119.8±79.6 minutes, with a median of 105 minutes. Duration of anesthesia was 177.6±110 minutes.

After analysis of the time interval between 7:00 am and the entrance of the first patient of the day in the OR in 990 operations, we obtained a mean of 1.2 hour. The first elective operation of the day began with a delay. The mean with standard deviation of the time interval between 7:00 am and the entrance of the first patient of the day into the OR was 71.7±34.3 minutes; the median was 72 minutes. The time interval varied from -50 to 153 minutes. This is because, in some specific situations, the patient entered the OR before 7:00 am, *e.g.*, at 6:10 am. Therefore, the minimum value recorded was -50 minutes.

**Figure 1:**
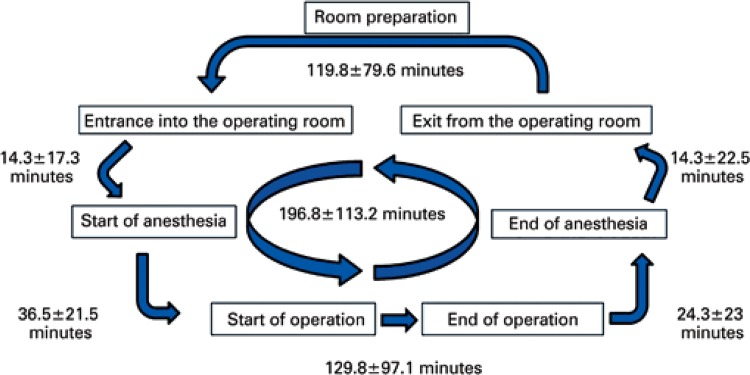
The graph shows the process in the operating room, indicators of time with the means and standard deviations (in minutes)

The indicators of OR time of the university hospital and the times of the respective intervals are displayed in figure 1.

## DISCUSSION

The operating center is a complex environment, unpredictable, dynamic, and available 24 hours a day. The origin of the patients is not the same, nor is their clinical condition. There are patients referred from the emergency room, intensive care unit, inpatients units, and who came from home, as well as elective, and urgency or emergency operations. After the procedure, the patients go to different units, are discharged home, OR units or to intensive care unit. The professionals who work in the sector have different characteristics that varies by specialty,* e.g.*, a surgery for cataracts correction and a heart transplantation have different logistics and are performed within the same environment. Additionally, different areas divide the responsibilities: information technology, hospital administration, pharmacy, and nursing. The primary focus should always be on the patient and on the physician responsible for him/her. Even with its great variability, management of the OR should be done in a dynamic and efficient manner, to avoid waste.^(13,14)^ We encountered lack of national data on this topic in the literature consulted, as information on management of the ORs, number of surgeries performed in the country and costs.

The appropriate data collect is fundamental to know the characteristics of the sector, identify weaknesses, correct them, and make it more efficient. It is an ongoing process for continuous improvement. The data collection in the OR is mandatory, and carried out by the nursing staff through a manually filled-in form. It was obtained 30% of incomplete data, or by blank fields of the form or due to illegible data. The collection system should be optimized and done in a digital way in a near future.

We analyzed more than 8,000 procedures carried out during a one-year period, with complete information. This allowed us to obtain mean durations of specific procedures. Despite the unforeseen factors and complications that can occur in a surgery, time management planning of the OR should be done based on this information.^(12,13)^ The duration of a surgery depends on the patient’s individuality, the unique characteristic of the disease, and the ability of the surgeon, but the mean duration of the procedure takes into account such variations. Preparation and cleaning of the OR do not depend on such eventualities and are a part of a process with little variation. In OR management, time needs to be divided into operative time and non-operative time.^(14,15)^

The mean time of duration of the operations was similar to the reference standard performance described in literature, *i.e.*, roughly 120 minutes.^(15)^ The difference appeared in the time between the entrance of the patient into the OR and the start of the operation; the mean time was 48 minutes. The literature recommends 19 minutes. The same occured with the mean of time between the end of the operation and the exit of the patient from the OR, 30 minutes *versus* 11 minutes.^(14-16)^ In the operative time indicators, we would have the potential of improving 48 minutes per procedure: [(48 – 19) + (30 – 11)].

The differences in non-operative time indicators were greater. The ideal time for OR preparation (turnover) between operations is up to 25 minutes.^(4,11,14,15)^ This interval starts counting with the exit of the patient from the OR and the entrance of the next patient, and it covers some actions, such as patient transport, nurses (circulating in the OR,) removal of surgical instruments, fill out forms, send biological materials – fluids, biopsies, surgical specimens, clean of the OR, and replacement of materials (surgical and anesthetic) for the next operation.

In our study, it was identified an interval of 119 minutes, that is, four times greater than the ideal reported in literature. The greater the idle OR time, the worse it is for the sector. The cost of opportunity is great, that is, an essential activity is not being carried out, with waste of the available resources.^(16-19)^

An idle OR in a highly complex sector is an insult to the patients that are on a waiting list for a vacancy for surgical treatment. The Brazilian Public Health System (SUS - *Sistema Único de Saúde*) is overburdened. The demand of patients is greater that the supply of treatments, and part of the cause is the inefficient OR.^(4,5)^

Although this study was conducted at a public hospital, considering the economic differences and the different objectives, private hospitals do not offer either an efficient OR. The delays and wastes are frequent.^(19)^ The comparison between the public and private systems is difficult, since one generates costs, the other generates revenue.

Unfortunately, we do not have national data on costs and expenses in the OR. The use of American literature is inevitable. In the United States, the average cost for a surgical procedure is estimated at US$ 15.00 to US$ 20.00 per minute (US$ 900.00 to US$ 1,200.00 per hour), not including medical fees. At least half of this value is fixed cost and the variable cost depends largely on how the professionals are paid.^(11,16)^ When we consider the idle time of an empty OR, the costs are higher; in the United States, each minute wasted can vary from US$ 60.00 to US$ 100.00.^(16)^

The determination of a value to charge a service provided in the OR varies with the complexity of the operation, the materials used, and the structure necessary. Some hospitals in São Paulo, (SP) (city) offer tables of values with managed medical procedures and the hour of OR varies from R$ 950,00 (R$ 15,83 per minute) to R$ 1.012,50 (R$ 16,87 per minute), not including fees of the surgeon and assistants.^(20,21)^

About the non-operative idle time, the mean time measured per OR was 155 minutes. As an exercise, we considered the cost of the idle minute as R$ 120,00 (US$ 60.00)^(16)^ (exchange rate US$ 1 = R$ 2,00) and thus, the waste of the idle OR would be R$ 18.600,00 (120 x 155). If we apply this value to the 8,420 surgeries performed in 2011, we would have a total wasted value of R$ 156.612.000,00 just in one year. In a private hospital of São Paulo (SP), a project to improve processes showed the value of the waste with delays in the OR: “…the 30-minute delays resulted in a R$ 300 million loss a year.”^(19)^ However, the time variable is more important than its cost. Resources can be substituted, replaced, and become obsolete, whereas time, when wasted, is lost forever.^(6,7,18)^ In our study, non-operative time was greater than the operative time.

Another important quality indicator that should be analyzed is the first operation of the day. This one should initiate at the scheduled time, since it is not subject to the unpredictability of previous procedures. The night shift staffs set up this OR, without succeed a used OR. Since these data require a specific calculation for each procedure, a random sample of the ORs was chosen, as well as for the turnover analysis. The delay of the first operation of the day was approximately 1.2 hour and was reflected in the sector’s organization. We consider this an important fact, since it is the intersection of the indicators of time, process, and result. It shows the logistics of the OR and the coordination among the multiprofessional and support areas, such as supplies/pharmacy, admission, patient transport, nursing staff, and physicians. If the first operation is delayed, there is a cascade effect difficult to be corrected throughout the day. All other subsequent operations will be delayed. Although sounds paradoxical, all efforts to reduce the time of preparation of the OR, such as improvement in patient transport, clean and set up the OR, can be done without hire extra personnel or acquiring new equipment.^(6,9,13)^ In the OR, the processes result primarily from methods and the human factor.^(4-6,10,22)^

There are two well-known methods used in administration and process improvement to adjust management of the OR: Six Sigma and Lean.^(6,7,9)^

The Six Sigma main philosophy is the reduction of variability. It is based on the assumption that each process should remain within acceptable limits. Its precept is to “do things correctly, without errors.”^(6,7,9)^ This method uses as a basis the following actions: Define, Measure, Analyze, Improve, and Control.^(6)^ One simple example of the application of this philosophy would be the prediction of time necessary to prepare the OR between scheduled operations. In general, scheduling is done in “full hours” − at 7:00 am, 9:00 am, 2:00 pm, etc. The ORs agenda does not foresee the time necessary for preparation between one operation and another. For example, the 7:00 am operation will last two hours and the next one will be scheduled for 9:00 am, instead of 9:20 am. With this, the day’s planning has already begun incorrectly. Another fact is to consider the actual average duration for the scheduled procedure and not rely only on the estimated duration made by professional who schedule the operation. For example, if the mean duration time of a lung lobectomy is 4 hours and the surgeon schedules it with an estimated duration of 3 hours, but it last 4 hours, the delay for subsequent operations is 60 minutes.

The quality management “Lean” focuses on remove waste and unnecessary steps processes. The “Lean” philosophy adopts a continuous improvement strategy for creating simple ways, direct to eliminate redundancies and excesses in a system. It also uses the customer perspective to define the quality. The main focus is to standardize production processes so, the flow can be optimized and all waste or inefficiencies removed.^(6,7,9)^

The OR is one of the convergence points of the hospital – intensive care unit, emergency room, and inpatients units, all depend on it. It is responsible for the greatest movement in admissions. A poorly managed OR creates problems for the entire hospital chain, with direct and indirect waste.^(22,23)^ There is waste in admissions when surgeries are cancelled, maintenance of patients in the intensive care unit, prolonged hospitalization, among others. This sector of the hospital should be highly qualified, with state-of-the-art equipment and skilled and prepared technical human factor for risk prevention and management. The environment needs to be prepared for error – this is the only way it can be avoided.^(24,25)^ One good analogy with aviation demonstrates that an operation needs preparation, planning, and constant vigilance.^(25)^

Since this topic is frequent and is present in most, if not in all Brazilian hospitals, further studies should be conducted to identify the causes and find new solutions.

## CONCLUSION

The collection of quality data is indispensable to prepare indicators and make correct decisions. This study displayed the dynamic process of an operating room. It showed how intervals between the non-surgical steps interfere in productivity and efficiency.

The indicators evaluated were lower than the performance considered satisfactory in the literature searched.
