# Supplementary material for: An assessment of the quality indicators of operative and non-operative times in a public university hospital
Source: Einstein (Sao Paulo). 2015 Oct-Dec;13(4):594–9. doi: 10.1590/S1679-45082015GS3289 (PMC4878636; doi:10.1590/S1679-45082015GS3289)
Supplement: Translation

# Translation: Avaliação dos indicadores de qualidade de tempo operatório e não operatório de um hospital universitário público

**Authors:** Altair da Silva Costa, Luiz Eduardo Villaça Leão, Maykon Anderson Pires de Novais, Paola Zucchi

**Affiliations:** 1 Hospital Israelita Albert Einstein, São Paulo, SP, Brasil.; 2 Universidade Federal de São Paulo, São Paulo, SP, Brasil.

**Keywords:** Indicadores de qualidade em assistência à saúde, Salas cirúrgicas, Duração da cirurgia

## Abstract

**Objetivo:**

Avaliar os indicadores de tempo do centro cirúrgico de um hospital universitário para melhoraria de sua eficiência.

**Métodos:**

Foi realizado um estudo descritivo transversal a partir da base de dados da tecnologia da informação do centro cirúrgico. A amostra foi obtida a partir de janeiro de 2011 a janeiro de 2012. Foram incluídas as operações realizadas em sequência na mesma sala cirúrgica, das 7 às 17h, eletivas ou de urgências. Os procedimentos com dados incompletos no sistema foram excluídos, assim como as operações depois das 17h ou realizadas em fins de semana ou feriados.

**Resultados:**

Foi medido o tempo operatório e não operatório de 8.420 operações realizadas. Os tempos operatórios (média e desvio padrão) de anestesia e de cirurgia foram 177,6±110 e 129,8±97,1 minutos, respectivamente. O tempo total do paciente em sala cirúrgica (média e desvio padrão) foi de 196,8±113,2. O tempo não operatório, por exemplo, entre a chegada do paciente e o início da anestesia, foi de 14,3±17,3 minutos. O tempo de preparo e entrada do próximo paciente na sala cirúrgica foi 119,8±79,6 minutos. O tempo não operatório total foi de 155 minutos.

**Conclusão:**

Atrasos frequentes ocorreram nas salas de cirurgia e tiveram um grande efeito sobre o fluxo de pacientes e a utilização de recursos. O tempo não operatório foi maior que o operatório. Portanto, é possível aumentar a capacidade do centro cirúrgico por meio da gestão e do treinamento dos profissionais envolvidos. Os indicadores oferecem uma ferramenta para melhorar a eficiência das salas de cirurgia.

## INTRODUÇÃO

Nos Estados Unidos, foram realizados 15 milhões de procedimentos cirúrgicos (495 procedimentos/10 mil habitantes), com custos hospitalares ao redor de US$ 171 bilhões. Os pacientes cirúrgicos têm um gasto maior que os clínicos, respectivamente, US$ 2,900,00 e US$ 1,400,00 por dia de internação. A sala cirúrgica deve ser eficiente e otimizada, bem como o tempo e os recursos disponíveis.^(1-3)^

O centro cirúrgico é uma das estruturas mais complexas do sistema hospitalar: funciona 24 horas por dia, sete dias da semana e existe uma diferença de complexidade dos equipamentos e procedimentos. Também necessita de diversas classes de profissionais – administrador, médicos, enfermagem, informática, farmácia, limpeza, entre outros. A logística de material deve ser perfeita, pois a ausência deste implica na qualidade do procedimento e no resultado. Além disso, temos a presença de material biológico e o risco de vida do paciente sempre presente.^(4,5)^

O centro cirúrgico também é a intersecção de vários setores do hospital, como terapia intensiva, enfermarias, pronto-socorro, laboratório e diagnóstico por imagem. O funcionamento adequado depende ainda da estrutura física, de novas tecnologias, de materiais e equipamentos adequados manuseados por mão de obra capacitada, treinada e competente.^(6,7)^ É um ambiente altamente diferenciado, por isso os desperdícios de tempo e material deveriam ser mínimos. Um centro cirúrgico pouco eficiente gera mais riscos e desperdícios ao hospital, aos pacientes e funcionários. O centro cirúrgico deveria ser o setor mais eficiente do hospital, com processos em constante controle e otimização.^(8)^

Os processos representam tarefas em execução, com relação direta ou indireta com o procedimento a ser realizado.^(9,10)^ Para mensurar os processos, são coletados dados de formulários e, assim, criados indicadores de produtividade, como, por exemplo, operações suspensas, atraso de profissionais, número de operações por sala cirúrgica ou por dia ou falta de material adequado na sala cirúrgica. Outros indicadores de tempo são utilizados nos serviços hospitalares e no centro cirúrgico como, por exemplo, tempo de anestesia e tempo para preparo da sala cirúrgica.^(11,12)^

O preparo da sala cirúrgica, o *turnover time*, engloba a retirada do paciente da sala cirúrgica, a limpeza, o preparo dos instrumentais e materiais necessários, a presença dos profissionais no local (enfermagem, médicos e técnicos) e a entrada do paciente na sala cirúrgica.^(4,10,13-15)^ O tempo ideal de preparo da sala cirúrgica entre uma operação e outra pode ser classificado como de alto desempenho se até 25 minutos; desempenho médio, se entre 25 e 40 minutos; desempenho sofrível, se maior que 40 minutos.^(4,10,13-15)^

O *Surgery Management Improvement Group*, de Michigan, Estados Unidos, publicou qual o tempo ideal de cada intervalo.^(14)^ Foi desenvolvido um programa para otimizar o tempo no centro cirúrgico e medido qual seria o impacto dele após sua implantação. Foi calculado o tempo útil de cada sala cirúrgica, o desempenho ideal e, assim, foi determinado o potencial de melhora. O tempo medido entre a entrada do paciente na sala cirúrgica e o início da incisão foi de 45,5 minutos. O desempenho ideal seria 19 minutos, e o potencial de melhora foi de 26,5 minutos por operação. Outro exemplo foi o preparo da sala cirúrgica entre um procedimento e outro; o tempo medido foi de 34,8 minutos, o desempenho ideal de 23,2 minutos, e o potencial de melhora foi de 11,6 minutos.^(14)^

A gestão do tempo no centro cirúrgico merece destaque. A organização (ou a falta dela), o custo de uma sala cirúrgica parada e o desgaste dos profissionais são exemplos de desperdícios de tempo e recursos acarretados ao paciente, aos familiares, ao hospital e aos profissionais.^(11,12)^

## OBJETIVO

Avaliar os indicadores de tempo do centro cirúrgico de um hospital universitário para melhorar a eficiência.

## MÉTODOS

Define-se “cirurgia” como o ramo da medicina que se dedica ao tratamento das doenças, lesões, ou deformidades, por processos manuais, denominados operações ou intervenções cirúrgicas. Cirurgia é uma habilidade a ser aprendida, estudada e ensinada. O tratamento e o ato em si são denominados “operação”. O cirurgião realiza uma operação e estuda ou ensina cirurgia.

Foi realizado um estudo descritivo transversal a partir da base de dados do sistema de tecnologia de informação do centro cirúrgico do hospital universitário da Universidade Federal de São Paulo. A amostra foi constituída por operações realizadas no período de janeiro de 2011 a janeiro de 2012. Foram incluídas intervenções cirúrgicas realizadas em sequência, nas mesmas salas cirúrgicas, durante o horário de rotina do centro cirúrgico, entre 7 e 17h, eletivas ou de urgências. Foram excluídos os procedimentos com dados incompletos no sistema, operações com início após às 17h ou realizadas nos finais de semana ou feriados, pois, nesses períodos, o quadro de funcionários era reduzido. O centro cirúrgico do hospital universitário era composto, na época, por 19 salas cirúrgicas ativas. O processo que ocorreu na sala cirúrgica foi dividido conforme as etapas a seguir para coleta das respectivas variáveis: 1) tempo do paciente em sala cirúrgica; 2) tempo da duração da anestesia; 3) tempo da duração da operação; 4) tempo entre a entrada do paciente na sala cirúrgica e o início da anestesia; 5) tempo entre o início da anestesia e o início da operação; 6) tempo entre o término da operação e o término da anestesia; 7) tempo entre o término da anestesia e a saída da sala cirúrgica; 8) tempo para entrada do próximo paciente na sala cirúrgica; (*turnover*) – amostra aleatória; 9) tempo entre 7h e entrada do primeiro paciente do dia – amostra aleatória. Para analisar estes dois últimos indicadores, o tempo de preparo da sala cirúrgica (*turnover*) e a entrada do primeiro paciente do dia na sala cirúrgica, obtivemos os dados por amostragem e realizamos cálculo individual de cada operação. Foram selecionadas, de maneira aleatória, seis salas cirúrgicas para obter uma representatividade de, no mínimo, 10% das operações.

A primeira operação do dia não estava sujeita à imprevisibilidade das outras e, por isso, era um indicador importante de tempo e processo. Refletia a organização do setor.

O projeto foi aprovado pelo Comitê de Ética e Pesquisa da instituição, número do parecer 165.292/2012, CAAE: 07233312.9.0000.5505, com autorização da coordenação do centro cirúrgico do hospital. Houve dispensa do Termo de Consentimento Livre e Esclarecido em razão do método de pesquisa aplicado.

## RESULTADOS

Foram realizados 12.114 procedimentos no período de janeiro de 2011 a janeiro de 2012. Destes, foram incluídas no estudo 8.420 (69,5%) operações e foram excluídos por dados incompletos 3.694 (30,5%) procedimentos. As operações eletivas foram 4.660 (55,3%) e as urgências, 3.760 (44,7%).

Para cálculo do *turnover *e da primeira operação do dia, com a seleção aleatória das seis salas cirúrgicas, analisamos 990 operações (11,7% do total).

A variação do tempo das operações foi, de zero minutos a 14,5 horas. A complexidade também foi muito diversa, incluindo desde procedimentos como retirada de cateter, ressecção de nevos, até transplantes. Praticamente todas as especialidades cirúrgicas fizeram parte dos procedimentos analisados: cirurgia cardíaca, vascular, neurocirurgia, cirurgia geral, gastrocirurgia, otorrinolaringologia, cabeça e pescoço, cirurgia torácica, ginecologia, oftalmologia, entre outras.

Na tabela 1 estão os tempos referentes à permanência do paciente na sala cirúrgica, anestesia e da operação em si.

**Tabela 1:** Tempo de permanência do paciente em sala cirúrgica, tempo de anestesia e tempo operatório de 8.420 procedimentos

	Média ± desvio padrão	Mediana	Mínimo-Máximo
Paciente em sala cirúrgica	196,8±113,2	177	12-900
Anestesia	177,6±110	159	3-897
Operação	129,8±97,1	111	0-843

Valores expressos em minutos.

Os indicadores de intervalo de tempo mostraram o que ocorre entre os procedimentos no tempo não operatório, quanto tempo foi necessário entre o término de uma etapa e início da outra (Tabela 2).

**Tabela 2:** Tempo entre as etapas da operação de 8.420 procedimentos

	Média ± desvio padrão	Mediana	Mínimo-Máximo
Entrada sala cirúrgica – início da anestesia	12,3±17,3	5	0-93
Início anestesia – início operação	36±21,5	39	0-180
Término operação – término anestesia	24,3±23	15	0-276
Término anestesia – saída sala cirúrgica	14,7±22,5	5	0-258

Valores expressos em minutos.

Das 990 operações analisadas, tivemos uma média de aproximadamente duas horas para preparo da sala cirúrgica (*turnover*) e entrada do próximo paciente, com tempo máximo de quase oito horas. A média com desvio padrão do intervalo de tempo entre a saída do paciente da sala cirúrgica e entrada do próximo paciente na sala cirúrgica foi de 119,8±79,6 minutos, com mediana de 105 minutos. A duração da anestesia foi de 177,6±110 minutos.

Após a análise do intervalo de tempo entre 7h e a entrada do primeiro paciente do dia na sala cirúrgica em 990 operações, obtivemos uma média de 1,2 hora. A primeira operação eletiva do dia já começava com atraso. A média com desvio padrão do intervalo de tempo entre 7h e entrada do primeiro paciente do dia na sala cirúrgica foi de 71,7±34,3 minutos; a mediana foi de 72 minutos. O intervalo de tempo variou de -50 a 153 minutos. Isso porque, em algumas situações específicas, o paciente entrou na sala cirúrgica antes das 7h, por exemplo, às 6h10. Por isso, o valor mínimo anotado foi de -50 minutos.

Os indicadores de tempo do centro cirúrgico do hospital universitário, e os tempos dos respectivos intervalos são mostrados na figura 1.

**Figura 1:** Esquema do processo na sala cirúrgica, indicadores de tempo com as médias e desvio padrão (em minutos)

## DISCUSSÃO

O centro cirúrgico é um ambiente complexo, imprevisível, dinâmico e disponível 24 horas por dia. A origem dos pacientes não é a mesma e nem a condição clínica. Há pacientes do pronto-socorro, da terapia intensiva, das enfermarias e do domicílio, bem como há operações eletivas, de urgência ou emergência. Após o procedimento, os pacientes também vão para locais diferentes, alta para casa, para o quarto ou terapia intensiva. Os profissionais que trabalham no setor têm características diferentes que variam com as especialidades, por exemplo, uma operação para correção de catarata e um transplante cardíaco têm logísticas diferentes realizadas no mesmo ambiente. Além disso, diferentes áreas dividem as responsabilidades: tecnologia da informação, administração hospitalar, farmácia e enfermagem, mas o foco principal sempre dever ser no paciente e no médico responsável por este. Mesmo com sua enorme variabilidade, a gestão do centro cirúrgico deve ser feita de forma dinâmica e eficiente, para evitar desperdícios.^(13,14)^ Encontramos uma falta de dados nacionais sobre esse tópico na literatura consultada. Informações sobre gestão de centro cirúrgico, número de operações realizadas no país e custos são escassos.

Aquisição de informação adequada é fundamental para conhecer as características do setor, identificar as falhas, corrigi-las e torná-lo mais eficiente. É um processo contínuo para melhoria contínua. Nossa coleta de informação no centro cirúrgico é feita de maneira obrigatória pela enfermagem por meio de um formulário preenchido à mão. Obtivemos 30% de dados incompletos, principalmente pela ausência dos dados em determinados campos do formulário ou por dados ilegíveis. O sistema de coleta deve ser otimizado e feito de maneira digital em um futuro próximo.

Analisamos aqui mais de 8.000 procedimentos realizados no período de um ano, com informações completas. Isso possibilitou obter médias da duração de procedimentos específicos. Apesar das imprevisibilidades e complicações que podem ocorrer numa operação, o planejamento da gestão do tempo no centro cirúrgico dever ser feito com base nessas informações.^(12,13)^ A duração de uma operação depende da individualidade do paciente, da característica singular da doença e da habilidade do cirurgião, mas a média da duração do procedimento contempla tais variações. O preparo e limpeza da sala cirúrgica não dependem de tais eventualidades e fazem parte de um processo com pouca variação. Na gestão do centro cirúrgico é necessária a divisão em tempo operatório e não operatório.^(14,15)^

A média do tempo de duração das operações foi semelhante ao desempenho padrão de referência descrito na literatura, ao redor de 120 minutos.^(15)^ A diferença aparece no tempo entre a entrada do paciente na sala cirúrgica e o início da operação; a média que encontramos foi de 48 minutos. A literatura recomenda 19 minutos. O mesmo ocorre com a média de tempo entre o término da operação e a saída do paciente da sala cirúrgica, 30 minutos contra 11 minutos.^(14-16)^ Nos indicadores de tempo operatório, teríamos o potencial de melhora de 48 minutos por procedimento: [(48 – 19) + (30 – 11)].

As diferenças nos indicadores de tempo não operatório foram maiores. O tempo ideal para preparo da sala cirúrgica (*turnover*) entre as operações é de até 25 minutos.^(4,11,14,15)^ Esse intervalo inicia sua contagem com a saída do paciente da sala cirúrgica e termina com a entrada do próximo, compreendendo ações como transporte dos pacientes, da enfermagem (circulante de sala cirúrgica), retirada de instrumentos cirúrgicos, preenchimentos de fichas, encaminhamento de insumos biológicos – líquidos, biópsias, peças cirúrgicas, limpeza da sala cirúrgica e reposição de materiais (cirúrgicos e anestésicos) para a próxima operação.

Em nosso estudo, constatamos um intervalo de 119 minutos, ou seja, 4 vezes maior que tempo ideal relatado na literatura. Quanto maior tempo de sala cirúrgica ociosa, pior para o setor. O custo da oportunidade é enorme, ou seja, deixa-se de produzir uma atividade essencial, com desperdício de recursos disponíveis.^(16-19)^

Uma sala cirúrgica parada em um setor altamente complexo é um ultraje aos pacientes que aguardam em listas de espera por uma vaga para tratamento cirúrgico. O Sistema Único de Saúde brasileiro está sobrecarregado. A demanda de pacientes é maior que a oferta de tratamentos e, parte da causa disto é um centro cirúrgico ineficiente.^(4,5)^

Apesar de este estudo ter sido realizado em um hospital público, considerando as diferenças econômicas e os objetivos diferentes, os hospitais privados não oferecem um centro cirúrgico com eficiência exemplar. Os atrasos e o desperdício também são frequentes.^(19)^ A comparação entre o sistema público e privado é difícil, pois enquanto um gera custo, o outro gera receita.

Infelizmente não dispomos de dados nacionais sobre custos e despesas no centro cirúrgico. A utilização da literatura americana é inevitável. Nos Estados Unidos, o custo médio para um procedimento cirúrgico é estimado entre US$ 15.00 a US$ 20.00 por minuto (US$ 900.00 a US$ 1,200.00 por hora), sem os honorários médicos. Pelo menos metade desse valor é de custo fixo e o custo variável depende, em grande parte, de como os profissionais são pagos.^(11,16)^ Quando consideramos o tempo ocioso de uma sala cirúrgica parada, os custos são maiores, nos Estados Unidos, cada minuto desperdiçado pode variar de US$ 60.00 a US$ 100.00.^(16)^

A determinação de um valor para cobrança de um serviço oferecido no centro cirúrgico varia com a complexidade da operação, material utilizado e estrutura necessária. Alguns hospitais da cidade de São Paulo (SP) oferecem tabelas com valores dos procedimentos médicos gerenciados e a hora da sala cirúrgica varia de R$ 950,00 (R$ 15,83 por minuto) a R$ 1.012,50 (R$ 16,87 por minuto), não inclusos os honorários do cirurgião e auxiliares.^(20,21)^

O tempo ocioso não operatório medido por sala cirúrgica por operação foi de 155 minutos. Para exercício, consideramos o custo do minuto ocioso em R$ 120,00 (US$ 60.00)^(16)^ (cotação US$ 1 = R$ 2,00) e assim, o desperdício da sala cirúrgica parada seria de R$ 18.600,00 (120 x 155). Se aplicarmos esse valor nas 8.420 operações de 2011, teríamos o valor total desperdiçado de R$ 156.612.000,00 apenas em um ano. Em um hospital privado de São Paulo (SP), um projeto de melhoria dos processos mostrou o valor do desperdício com os atrasos no centro cirúrgico: “os atrasos de 30 minutos resultavam em R$ 300 milhões de prejuízo ao ano.”^(19)^ Entretanto, a variável tempo é mais importante que custo. Recursos podem ser substituídos, repostos e tornam-se obsoletos, já o tempo, quando desperdiçado, perde-se para sempre.^(6,7,18)^ Em nosso estudo, o tempo não operatório foi maior que o tempo operatório.

Outro indicador de qualidade que deve ser analisado em destaque é a primeira operação do dia. Esta deveria iniciar no horário previsto, pois não está sujeita à imprevisibilidade dos procedimentos anteriores. Esta sala cirúrgica é montada pela equipe do turno da noite, sem suceder uma sala cirúrgica utilizada. Foi optado por uma amostra aleatória de salas cirúrgicas, pois estes dados exigiram um cálculo específico para cada procedimento, bem como a análise do *turnover*. O atraso na primeira operação do dia foi de aproximadamente 1,2 hora e refletiu na organização do setor. Consideramos este um dado importante, pois é a intersecção dos indicadores de tempo, processo e resultado. Ele mostra a logística do centro cirúrgico e a coordenação entre os multiprofissionais e áreas de apoio, como suprimentos/farmácia, internação, transporte de paciente, enfermagem e médicos. Se a primeira operação atrasa, ocorre um efeito em cascata na sala cirúrgica que dificilmente será corrigido no decorrer do dia. Todas as outras operações subsequentes atrasam.

Por mais paradoxal que pareça, todos os esforços para reduzir o tempo de preparo da sala cirúrgica, como a melhoria do transporte de pacientes, limpeza e montagem de sala cirúrgica, podem ser feitos sem a contratação de pessoal adicional ou a aquisição de novos equipamentos.^(6,9,13)^ No centro cirúrgico, os processos são decorrentes principalmente dos métodos e do fator humano.^(4-6,10,22)^

Para adequar as gestões do centro cirúrgico, existem dois métodos utilizados em gerenciamento e melhoria de processos bem conhecidos: Six Sigma e Lean.^(6,7,9)^

A filosofia Six Sigma tem como objetivo principal, a redução da variabilidade. Baseia-se no pressuposto de que cada processo deve estar dentro de limites aceitáveis. Tem como preceito “fazer as coisas direito, sem erros”.^(6,7,9)^ Esse método utiliza como base as seguintes ações: Definir, Medir, Analisar, Melhorar e Controlar.^(6)^ Um simples exemplo da aplicação dessa filosofia seria a previsão do tempo necessário para preparo das sala entre as operações agendadas. De um modo geral, os agendamentos são feitos em “horas cheias” − às 7h, 9h, 14h etc. A agenda do centro cirúrgico não prevê o tempo necessário de preparo entre uma operação e outra. Por exemplo, a operação das 7h terá duração de duas horas e a próxima será agendada às 9h ao invés das 9h20. Com isso, o planejamento do dia já começa errado. Outro fato é considerar a média real de duração para o procedimento agendado e não confiar somente na estimativa do profissional que agenda a operação. Por exemplo, se a média de duração de uma lobectomia pulmonar é de 4 horas, o cirurgião agenda com duração estimada de 3 horas, e dura 4 horas, isso gera um atraso de 60 minutos nas operações subsequentes.

A gestão da qualidade “Lean” se concentra na remoção de desperdícios e passos desnecessários de processos. A filosofia “Lean” adota uma estratégia de melhoria contínua para a criação de caminhos simples, diretos, para eliminar redundâncias ou exageros em um sistema. Também utiliza a perspectiva do cliente para definir a qualidade. O enfoque principal é a padronização de processos de produção para que o fluxo possa ser otimizado e todos os desperdícios ou ineficiências removidos.^(6,7,9)^

O centro cirúrgico é um dos pontos de convergência do hospital – dele dependem a terapia intensiva, pronto-socorro e enfermarias. É responsável pelo maior movimento de internações. Um centro cirúrgico mal gerenciado cria problemas em toda a cadeia hospitalar, com desperdícios diretos e indiretos.^(22,23)^ Há desperdício de internações quando temos operações suspensas, manutenção do paciente na unidade de terapia intensiva, internação prolongada, entre outros. Esse setor do hospital deve ser altamente qualificado, com equipamentos de ponta e fator humano técnico capacitado e preparado, para prevenção e gerenciamento de riscos. O ambiente precisa ser preparado para o erro e só assim o evitaremos.^(24,25)^ Uma feliz analogia com a aviação demostra que uma operação necessita de preparação, planejamento e vigilância constante.^(25)^

Uma vez que o assunto é frequente e está presente na maioria, senão na totalidade dos hospitais brasileiros, mais estudos devem ser realizados para identificar as causas e encontrar novas soluções.

## CONCLUSÃO

A coleta de informações de qualidade é imprescindível para formar indicadores e para tomada de decisão correta. Este estudo retratou um processo dinâmico de um centro cirúrgico. Ficou evidente como os intervalos entre as etapas não cirúrgicas interferem na produtividade e eficiência.

Os indicadores avaliados ficaram abaixo do desempenho considerado satisfatório na literatura consultada.
